# Integrated molecular and serological survey of *Rhodococcus equi* in horses from three regions of Kazakhstan

**DOI:** 10.3389/fvets.2025.1650186

**Published:** 2025-10-21

**Authors:** Makpal Zanilabdin, Gulnaz Ilgekbayeva, Bauyrzhan Otarbayev, Raikhan Nissanova, Gulzhan Mussayeva, Shinji Takai, Yasunori Suzuki, Tsutomu Kakuda, Serikzhan Kurman, Yerken Kassymov, Bayan Valiyeva

**Affiliations:** 1 Kazakh National Agrarian Research University, Faculty of Veterinary and Zooengineering, Almaty, Kazakhstan; 2 Virology Laboratory, Kazakh Scientific Research Veterinary Institute, Almaty, Kazakhstan; 3 National Veterinary Reference Center, Almaty, Kazakhstan; 4 School of Veterinary Medicine, Kitasato University, Towada, Japan; 5 Tokyo University of Agriculture and Technology, Fuchu, Japan

**Keywords:** *Rhodococcus equi*, seroprevalence, Kazakhstan, zoonotic pathogen, phylogenetic analysis, equine respiratory disease, molecular epidemiology

## Abstract

**Introduction:**

*Rhodococcus equi* is a facultative intracellular pathogen causing bronchopneumonia in foals; data from Central Asia are limited. We conducted a cross-sectional serological and molecular survey in horses from three regions of Kazakhstan (Kyzylorda, Almaty, Akmola).

**Methods:**

Sera from 312 animals (272 adults, 40 foals) on 20 farms were tested by indirect ELISA. Selected clinical samples underwent culture, PCR, and 16S rRNA sequencing.

**Results:**

Overall seroprevalence was 8.3% (26/312; 95% CI 5.8–11.9). Positivity among foals was 25.0% (10/40; 95% CI 14.2–40.2) versus 5.9% (16/272; 95% CI 3.7–9.3) in adults, with farm-level clusters observed in the Almaty region. *R. equi* was isolated from three foals; a representative sequence was deposited (GenBank OP448586).

**Discussion:**

Phylogenetic analysis placed the Kazakhstani isolate within a clade of equine-associated *R. equi* strains reported from Europe and East Asia (>99.5% identity). We provide molecularly confirmed evidence of *R. equi* circulation in horses from three regions of Kazakhstan, with higher seropositivity in foals and focal farm-level clustering. Findings support the need for broader geographic sampling, test validation against reference sera, and incorporation of management/risk-factor data. Limitations include the regional scope, small number of foals, and absence of environmental or human sampling.

## 1 Introduction

*Rhodococcus equi* is a facultative intracellular actinomycete of major veterinary relevance and recognized importance for immunocompromised human hosts (1). It is the principal etiological agent of pyogranulomatous bronchopneumonia in foals, especially during the first 6 months of life (2). Intracellular survival within alveolar macrophages, mediated by plasmid-encoded virulence-associated proteins, notably *VapA* - underpins pathogenicity and environmental persistence (3, 4). Reports of severe pulmonary and disseminated infections in immunocompromised patients contextualize *R. equi* within a One Health framework, without implying direct assessment here (5).

The global distribution of *R. equi* has been documented with endemic circulation reported in the United States (6), Canada (7), Australia (8), and several European countries (9). In North America, early detection and treatment aim to reduce mortality, the practice has been linked to antimicrobial resistance in North America (10). In Japan, birth month is associated with tracheal colonization in foals, consistent with seasonally modulated risk (11). Ecologically, *R. equi* is favored by warm, dry climates and alkaline soils; transmission is primarily via inhalation of contaminated dust, to which immunologically immature foals are particularly susceptible (10, 12, 13).

Notwithstanding advances in ecology and pathogenesis, substantial geographic gaps remain in prevalence estimates, molecular diversity, and epizootiological patterns (14). Central Asia - and Kazakhstan in particular - has been under-represented (15). Given a large national horse population (see Supplementary Figure S1), managed extensively across semi-arid rangelands, ecological and husbandry conditions plausibly favor persistence and dissemination of *R. equi* (16). However, systematic seroepidemiology and molecular characterization have been limited, constraining region-specific prevention, diagnostic, and control strategies.

An integrated approach is therefore warranted. Indirect ELISA can capture herd-level exposure but does not differentiate environmental contact from active infection (17). Microbiological isolation with molecular confirmation - e.g., 16S rRNA sequencing and plasmid-associated virulence profiling - enables verification of infection and phylogeographic inference. Few studies have combined these complementary modalities in settings with scarce baseline data, limiting comprehensive inference.

To address these gaps, we conducted an integrated serological and molecular investigation of *R. equi* in equine populations from three regions of Kazakhstan. We screened sera from 312 horses on 20 farms by indirect ELISA and, in parallel, undertook microbiological isolation, PCR-based confirmation, and 16S rRNA sequencing of selected isolates, followed by phylogenetic analysis against global references. Clinical observations in affected foals were recorded to contextualize laboratory findings. Collectively, these data provide initial, region-resolved evidence for *R. equi* circulation and a foundation for expanded studies incorporating assay validation and risk-factor assessment within a One Health context.

## 2 Materials and methods

### 2.1 Study design and sampling sites

**Figure 1:**
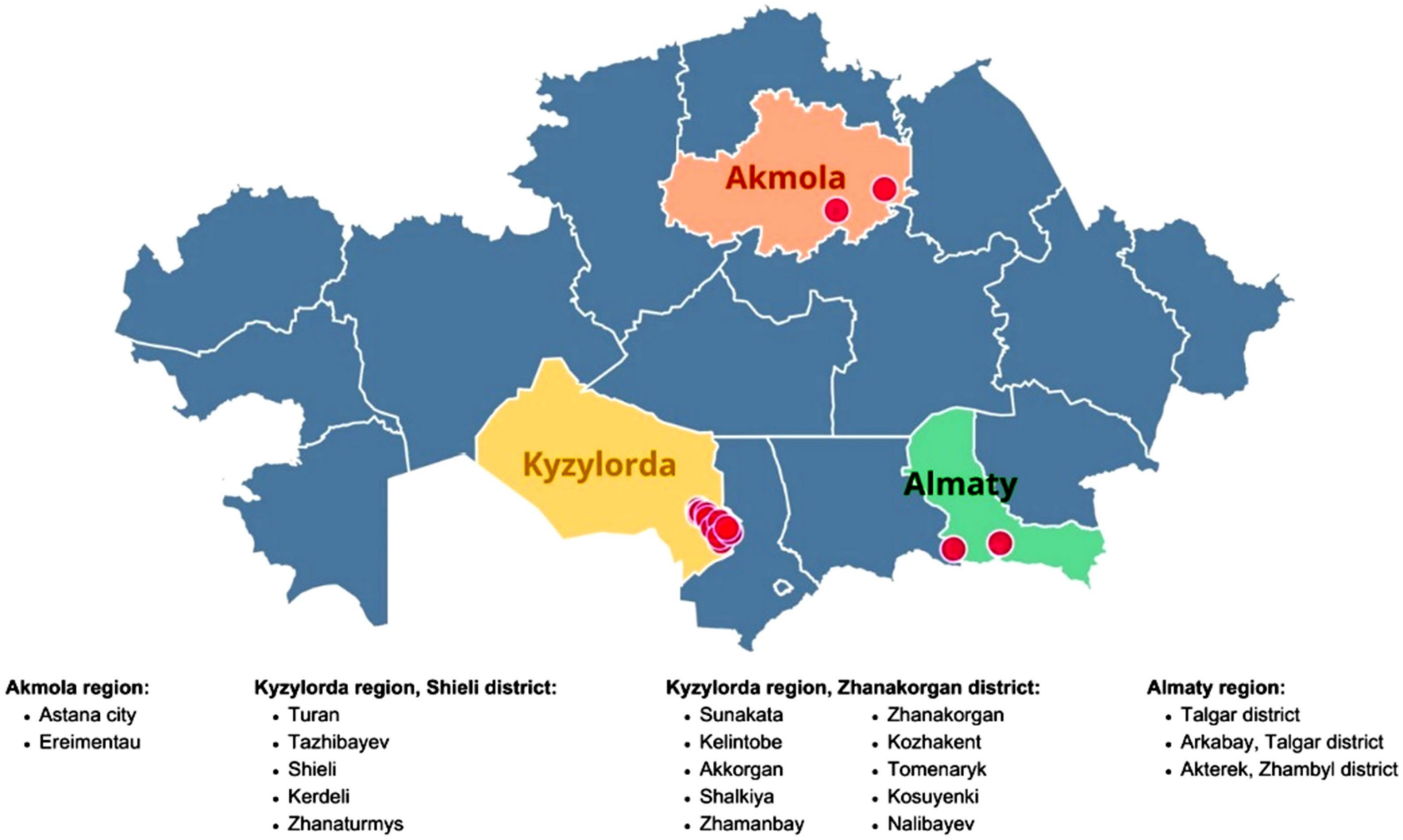
Geographic distribution of sampling sites for the serological and molecular survey of *Rhodococcus equi* in horses from three regions of Kazakhstan.

A cross-sectional study was conducted from March to September 2024 across three administrative regions of Kazakhstan (Figure 1): Kyzylorda (Shieli and Zhanakorgan districts), Almaty (Talgar and Zhambyl districts), and Akmola (Astana city and Ereimentau). Twenty horse-breeding farms were selected considering horse density, reported foal respiratory morbidity, and management practices. Sampling was stratified by age group (foals, adults) with farm-level clustering pre-specified for analysis. An *a priori* sample-size calculation *n* = [*Z*^2^·*p*(1–*p*)]/d^2^; *Z* = 1.96, *p* = 0.10, *d* = 0.05 indicated *n* ≈ 139; the realized sample (*n* = 312) exceeded this minimum.

### 2.2 Animal selection and clinical observations

We enrolled 312 horses (40 foals aged 1–6 months; 272 adults) using stratified sampling by age within farms. Inclusion criteria comprised clinically healthy animals and foals with respiratory signs (cough, nasal discharge, dyspnea, fever). A standardized veterinary examination form captured clinical status, age, sex, and farm-level variables at sampling.

### 2.3 Blood collection and serum preparation

Peripheral blood (10 mL, jugular venipuncture; Vacutainer, BD) was transported refrigerated (4 °C), centrifuged (3,000 rpm, 10 min), and sera aliquoted into sterile microtubes and stored at −20 °C until testing.

### 2.4 ELISA antigen preparation and serological testing

ELISA antigen was prepared from *R. equi* ATCC 6939 as previously described with minor modifications (18). Briefly, colonies from brain–heart infusion agar (38 °C, 5 days) were extracted in 0.0125 M sodium phosphate buffer (pH 7.4, 0.1% Tween 20), clarified (20,000 × g, 30 min, 4 °C), and adjusted to 1.0 μg/mL total protein in carbonate–bicarbonate buffer (pH 9.6) to coat plates overnight (4 °C). Heat-inactivated sera (56 °C, 30 min) were applied; bound IgG was detected with HRP-conjugate and TMB, read at 492 nm. The ELISA cut-off and seropositivity threshold were predefined as OD ≥ 0.3, corresponding to the mean OD of negative controls + 3 SD. We note that, in the absence of a validated reference (e.g., virus-neutralization), this threshold is epidemiologically motivated rather than ROC-optimized, and assay sensitivity/specificity were not estimated; formal test validation against a reference serum panel is planned in follow-up work.

### 2.5 Isolation and identification of *Rhodococcus equi*

Lung tissue, tracheobronchial lymph nodes, and nasal swabs were aseptically collected from seven foals with clinical signs consistent with rhodococcosis (respiratory distress and, in some cases, fatal outcome) at three farms, including sites in Talgar and Zhambyl districts (Almaty region). Each specimen represented a different foal: five clinically affected live foals contributed one nasal swab each, and two foals found dead contributed necropsy tissues (one lung and one tracheobronchial lymph node). Samples were inoculated onto Columbia blood agar and meat-peptone agar and incubated aerobically at 37 °C for 48–72 h. Presumptive *R. equi* colonies (mucoid, salmon-pink) were selected for further evaluation. Gram staining and biochemical profiling supported presumptive identification: catalase-positive, urease-positive, and a synergistic CAMP reaction with *Staphylococcus aureus*, consistent with the established phenotype of *R. equi*. Confirmed colonies were preserved for subsequent molecular characterization.

### 2.6 Molecular confirmation and sequencing

Genomic DNA was extracted from pure bacterial colonies using the GenUP™ Bacteria DNA Kit (Biotechrabbit, Germany) according to the manufacturer’s protocol. The 16S rRNA gene was amplified by PCR using universal bacterial primers 8F and 806R (8F, 5′-AGAGTTTGATCCTGGCTCAG-3′; 806R, 5′-GGACTACCAGGGTATCTAAT-3′), amplifying an ~800-bp region. PCR products were verified by 1.5% agarose gel electrophoresis and purified using a commercial PCR purification kit to remove unincorporated primers and nucleotides. Sanger sequencing was performed on an ABI 3500 Genetic Analyzer (Applied Biosystems, United States). Chromatograms were inspected and sequences trimmed for quality, then aligned using Clustal Omega and queried by NCBI BLASTn. Taxonomic identity was confirmed by ≥99% sequence similarity to validated *R. equi* strains in GenBank.

### 2.7 Phylogenetic analysis

To assess genetic relatedness with globally characterized strains (17), we performed phylogenetic analysis of 16S rRNA sequences. The consensus sequence from the Kazakhstani isolate was aligned with 25 reference *R. equi* sequences from GenBank spanning diverse geographies and hosts. Multiple sequence alignment used Clustal Omega; phylogeny was inferred by neighbor-joining in MEGA X with 1,000 bootstrap replicates. Evolutionary distances were computed using the Kimura two-parameter model. Trees were visualized and annotated to emphasize the placement of the Kazakhstani sequence relative to international isolates, facilitating inference on phylogeographic relationships and regional clustering.

### 2.8 Statistical analysis

We estimated the *a priori* sample size using *n* = [*Z*^2^·*p*(1–*p*)]/d^2^ (*Z* = 1.96 for 95% CI; *p* = 0.10; *d* = 0.05), yielding *n* ≈ 139. The realized sample (*n* = 312; 40 foals, 272 adults) exceeded this minimum; however, the age-group imbalance reduces power for age-stratified comparisons.

Because ELISA optical density (OD) values were non-normally distributed, we summarized OD as mean ± SD and used non-parametric tests: Kruskal–Wallis for multi-farm/region comparisons and Mann–Whitney U for foal–adult contrasts. Seropositivity was predefined as OD ≥ 0.3 (mean of negatives + 3 SD). In the absence of a validated reference (e.g., virus-neutralization), this threshold is epidemiologically motivated rather than ROC-optimized; formal assay validation against a reference serum panel is planned in follow-up work. Differences in seroprevalence across farms and regions were assessed by Chi-square or the Fisher exact test, as appropriate. For multivariable analyses, OD values were categorized (<0.3, 0.3–0.5, 0.5–1.0, ≥1.0) and multinomial logistic regression was fitted to evaluate associations with age, region, and herd size, using robust standard errors clustered by farm to account for intra-farm correlation. As sensitivity analyses, we considered generalized estimating equations (GEE) with an exchangeable correlation structure and mixed-effects logistic regression with a random intercept for farm. Predictor effects are reported as odds ratios (OR) with 95% confidence intervals. Analyses were conducted in R 4.3.1 (R Foundation), with *α* = 0.05. Where *p*-values did not meet the pre-specified significance threshold (*α* = 0.05), we refrain from inferring “trends” and emphasize point estimates with 95% confidence intervals.

### 2.9 Ethical approval

All animal procedures complied with institutional and national guidelines. The protocol was approved by the Local Ethics Committee for Animal Experiments, Kazakh National Agrarian Research University (Approval No. 02–2022, 22 November 2022). Written informed consent was obtained from all farm owners prior to sampling. Inclusion criteria comprised clinically healthy animals and foals with respiratory signs (cough, nasal discharge, dyspnea, fever). A standardized veterinary examination form was used to capture clinical status, age, sex, and farm-level variables at the time of sampling.

## 3 Results

### 3.1 Descriptive seroprevalence data

Sampling was conducted across three regions of Kazakhstan - Kyzylorda (Shieli, Zhanakorgan), Almaty (Talgar, Zhambyl), and Akmola (Astana, Ereimentau) - covering 20 farms (see Methods, Figure 1). In total, 312 sera (272 adults; 40 foals aged 1–6 months) were tested by indirect ELISA. Overall seroprevalence was 8.3% (26/312; 95% CI 5.8–11.9); foals showed 25.0% (10/40; 95% CI 14.2–40.2) versus adults 5.9% (16/272; 95% CI 3.7–9.3). The wide 95% confidence interval for foals reflects the smaller sample size (*n* = 40), limiting precision.

Seropositivity was not evenly distributed across farms. Farms 17 and 18 (Almaty region) exhibited the highest rates among foals—31% (4/13) and 46% (6/13), respectively; no seropositive foals were detected at Farm 16. Among adults, sporadic positives occurred at eight of the 17 farms, with most OD values near the cut-off, consistent with past exposure. A complete summary of seroprevalence, sample sizes, and OD ranges across all farms is provided in Table 1. The distribution of samples across predefined ELISA OD ranges (0.0–0.3; 0.3–0.5; 0.5–1.0; ≥1.0) is shown in Supplementary Figure S3. Farm-level mean OD with 95% confidence intervals are provided in Supplementary Table S1. Across farms, OD distributions did not differ globally (Kruskal–Wallis *p* = 0.3499).

**Table 1:** Distribution of the ELISA OD values of 312 horses at 20 horse-breeding farms in Kazakhstan.

Farm	Location	No. of horses	ELISA OD				Average ± SD		Range	
			<0.3	0.3–0.5	0.5–1.0	>1.0			Min	Max
1	KS1 Turan	20	18	2	0	0	0.140 ±	0.069	−0.032	0.342
2	KS Tazhibayev	20	19	0	1	0	0.067 ±	0.073	−0.124	0.542
3	KS Shieli	20	20	0	0	0	0.083 ±	0.079	−0.011	0.245
4	KS Kerdeli	20	19	1	0	0	0.144 ±	0.098	−0.123	0.342
5	KS Zhanaturmys	20	18	2	0	0	0.050 ±	0.125	−0.119	0.373
6	KZ*2 Sunakata	10	10	0	0	0	0.018 ±	0.077	−0.022	0.213
7	KZ Kelintobe	10	19	0	0	0	0.084 ±	0.043	−0.018	0.113
8	KZ Akkorgan	10	10	0	0	0	0.004 ±	0.035	−0.101	0.066
9	KZ Shalkiya	10	9	1	0	0	0.111 ±	0.063	−0.011	0.321
10	KZ Zhamanbay	10	9	1	0	0	0.187 ±	0.056	0.053	0.326
11	KZ Zhanakorgan	10	10	0	0	0	0.081 ±	0.018	0.050	0.128
12	KZ Kozhakent	10	10	0	0	0	0.126 ±	0.055	0.027	0.223
13	KZ Tomenaryk	10	5	5	0	0	0.256 ±	0.081	−0.002	0.370
14	KZ Kosuyenki	10	10	0	0	0	0.039 ±	0.044	−0.032	0.168
15	KZ Nalibayev	10	10	0	0	0	0.061 ±	0.064	−0.133	0.105
16	AZ3 Akterek	14	14	0	0	0	0.015 ±	0.024	−0.048	0.059
17	Almaty Talgar	13	9	0	1	3	0.432 ±	0.544	0.071	2.192
18	AZ Arkabai	13	7	3	2	1	0.469 ±	0.429	0.033	2.282
19	Astana city	44	41	2	1	0	0.071 ±	0.092	−0.050	0.661
20	Akmola Ermentau	28	28	0	0	0	−0.004 ±	0.039	−0.109	0.103
Total		312	286	17	5	4	0.110 ±	0.239	−0.124	2.282

These data indicate localized clusters of increased *R. equi* exposure, particularly among foals, which may reflect environmental contamination, herd management factors, or seasonal dynamics affecting bacterial transmission. The overall OD distribution was right-skewed (median 0.054; mean 0.110; IQR 0.006–0.141; min − 0.133; max 2.282), consistent with a largely seronegative population; see Supplementary Figure S2.

### 3.2 Statistical comparison of antibody response profiles

**Figure 2:**
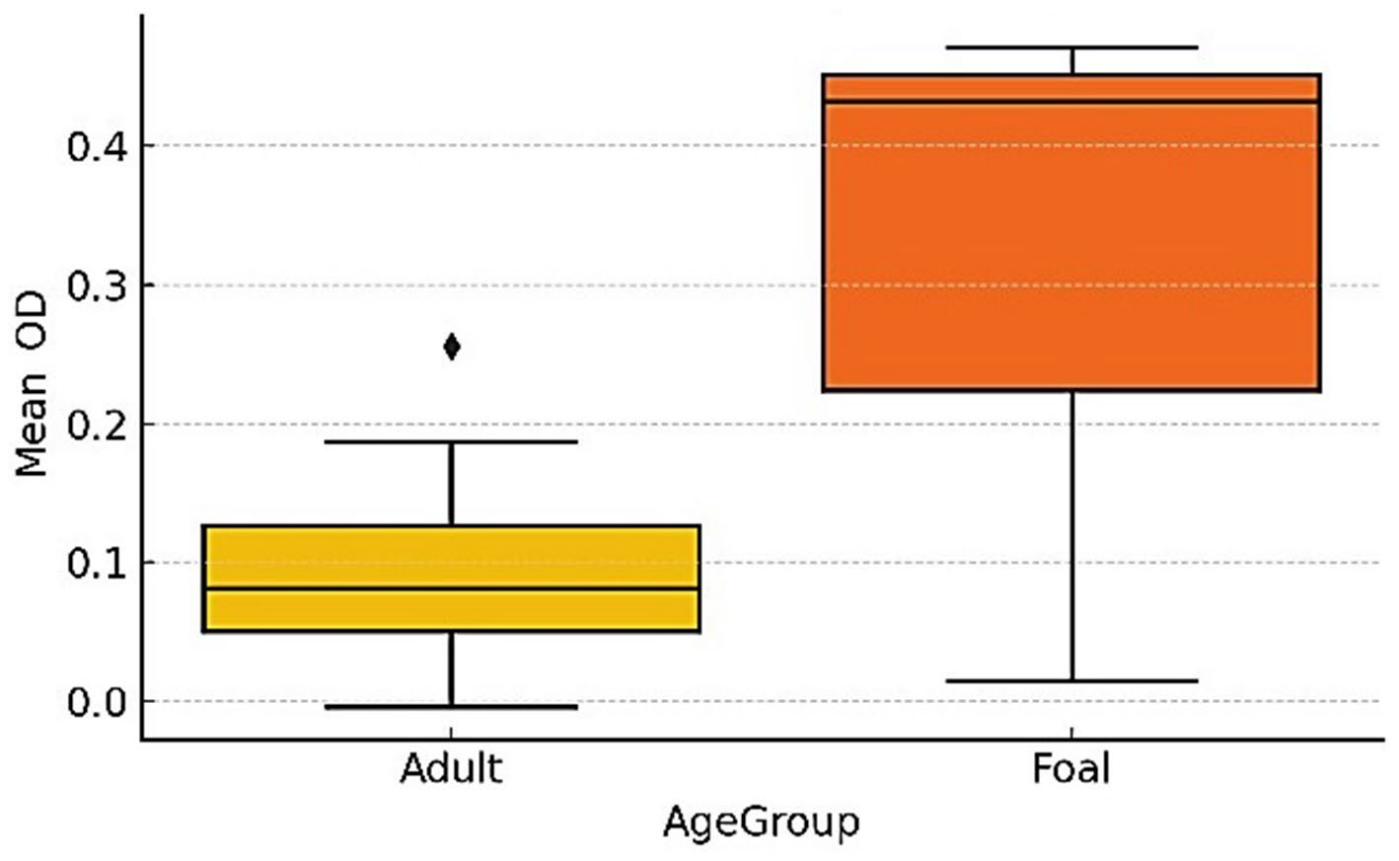
Comparative distribution of ELISA optical density (OD) values in foals and adult horses. Boxplots show the median (horizontal line), interquartile range (box), and minimum–maximum values (whiskers). The diamond (♦) represents an outlier. Statistical comparison between groups was performed using the Mann–Whitney U test (*p* = 0.305); no significant difference was detected (n.s.). Y-axis range was limited to 0.5 for clarity.

Comparison of antibody response distributions revealed a significantly higher likelihood of seropositivity among foals compared to adult horses. Foals exhibited 5.3-fold increased odds of testing seropositive (Fisher’s exact test, *p* < 0.001), consistent with heightened susceptibility and recent exposure to *R. equi*. While the Mann–Whitney U test did not show a significant difference in median OD values between age groups (*p* = 0.305), descriptively more high-OD values (>1.0) were observed in foals (Figure 2).

Median OD values did not differ significantly between age groups (Mann–Whitney U, *p* = 0.305); foals showed greater dispersion with several high-OD observations (>1.0). We therefore interpret group differences using point estimates and 95% confidence intervals; the sample-size imbalance (40 foals vs. 272 adults) limits power. This is consistent with age-related susceptibility described for *R. equi* in endemic regions, but causal inference is beyond the scope of these data.

**Figure 3:**
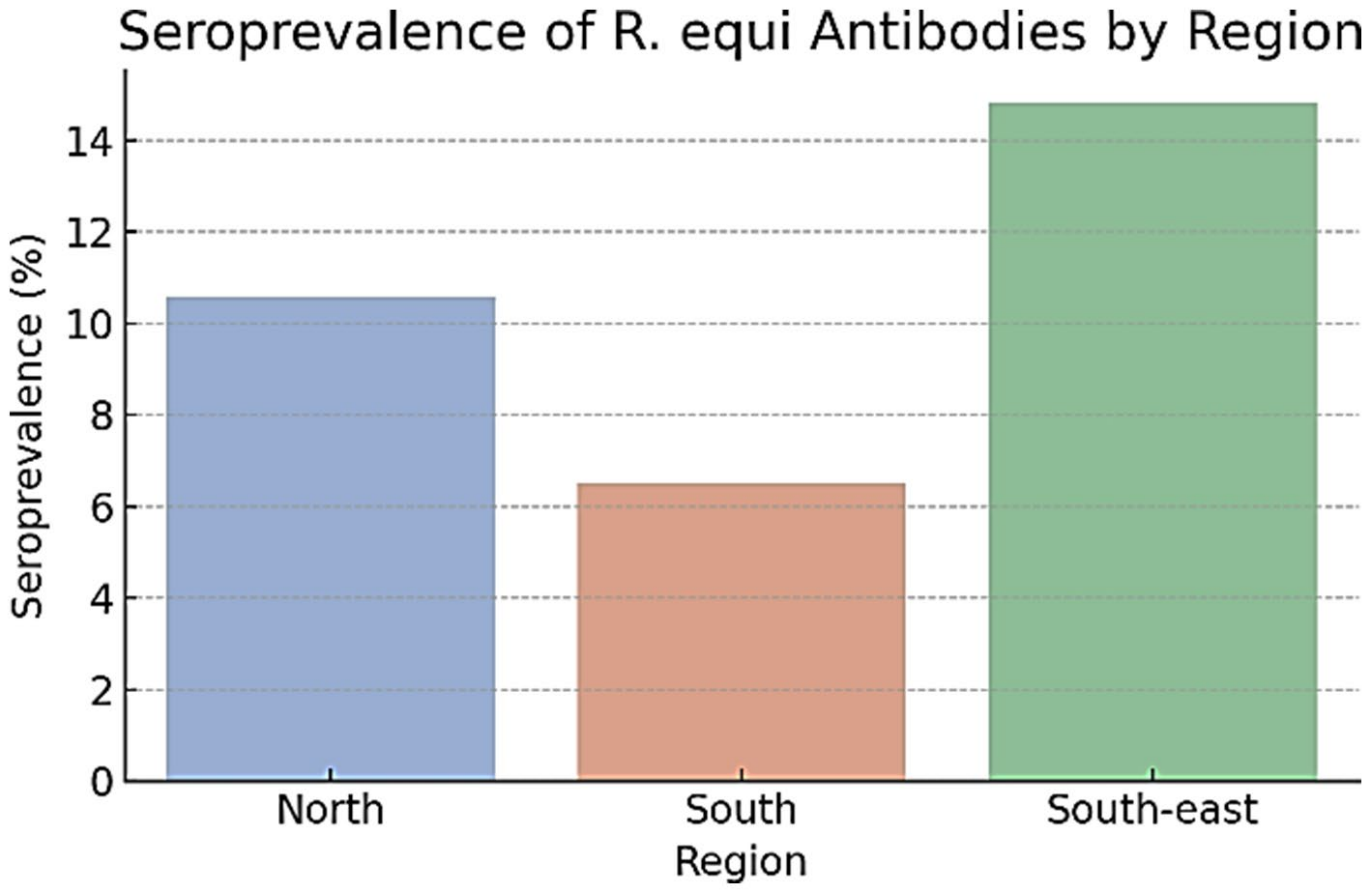
Seroprevalence of *R. equi* antibodies in horses by region of Kazakhstan.

The analysis determined regional *R. equi* antibody prevalence through the percentage of animals testing seropositive (OD ≥ 0.3). The highest seroprevalence was observed in the South-East region (14.8%), followed by the North (10.6%) and South (6.5%). While point estimates varied across regions, confidence intervals overlapped and no significant differences were detected (Kruskal–Wallis H = 0.036, *p* = 0.982; Figure 3).

Despite visible differences in prevalence rates, statistical analysis using the Kruskal–Wallis test indicated no significant variation in mean OD values across the three regions (H = 0.036, *p* = 0.982), suggesting that geographical factors alone may not fully explain observed exposure patterns.

### 3.3 Clinical case observation of a foal with suspected rhodococcosis

Across seven clinically affected foals examined at three farms, common signs included respiratory distress, profuse diarrhea, bilateral corneal opacity, and carpal/hock joint swelling; two foals were found dead. As a representative example, during field investigations, we documented a clinically significant case in a ~ 3-month-old foal from a breeding farm in Talgar District (Almaty region). The foal showed marked emaciation, profuse diarrhea, bilateral corneal opacity, and carpal/hock joint swelling - findings compatible with disseminated infection in foals, including disease caused by *R. equi*. Because culture was not obtained from this animal, microbiological confirmation was unavailable. The foal’s serum showed a high ELISA optical density (>2.0), far exceeding the predefined cut-off (OD ≥ 0.3), which supports recent/ongoing exposure; given that the ELISA is not a validated diagnostic test, these results should be interpreted as supportive rather than definitive.

Although culture was not obtained for this case, the high-titer antibody response together with the clinical presentation suggests a pathophysiologically relevant process consistent with *R. equi*; however, alternative etiologies cannot be excluded.

**Figure 4:**
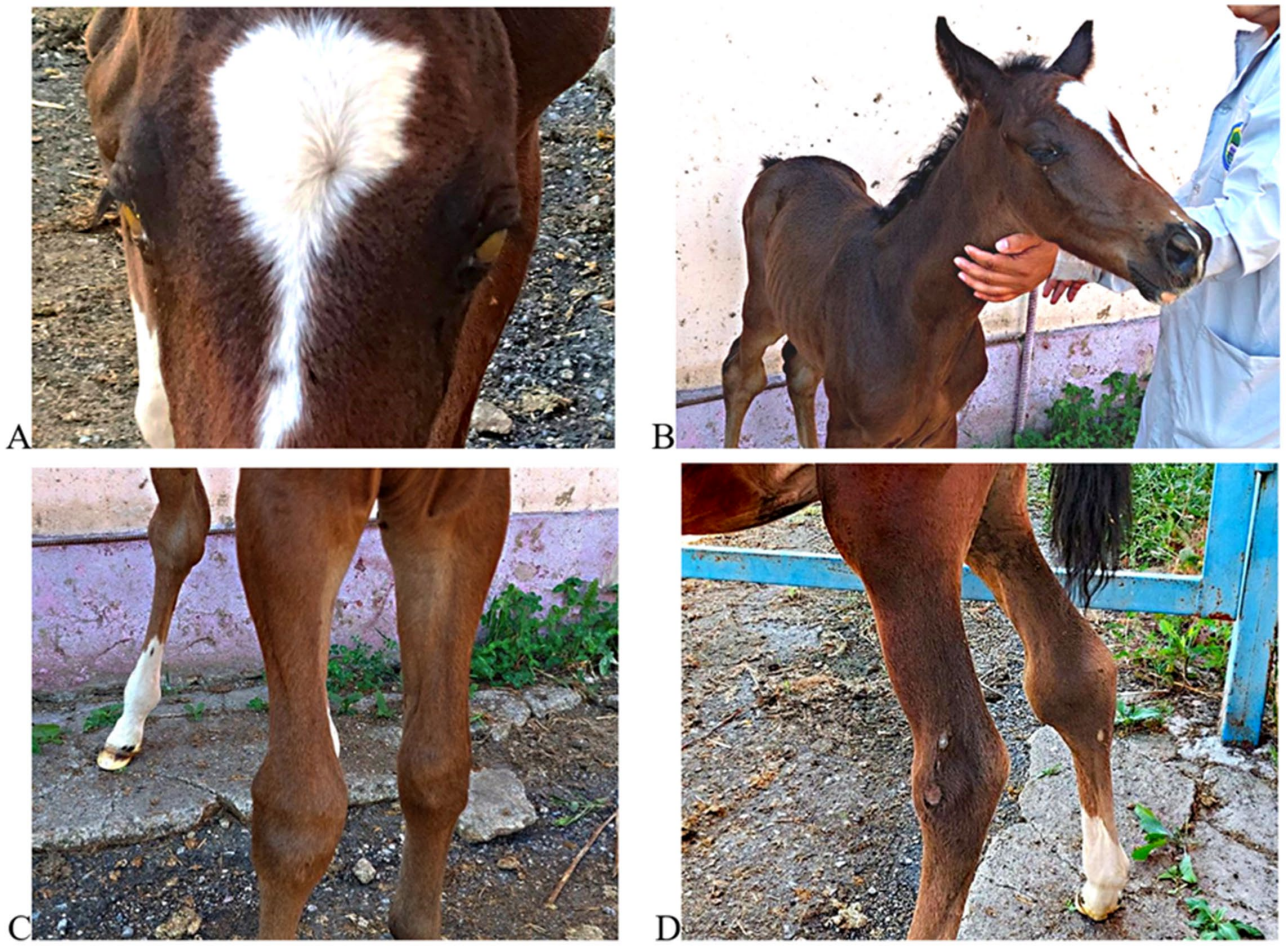
Clinical manifestations in a foal with suspected *Rhodococcus equi* infection. **(A)** Bilateral corneal opacity with yellowish exudate; **(B)** generalized emaciation and poor condition; **(C)** swelling of the left carpal joint; **(D)** bilateral hock joint enlargement. These images illustrate clinical signs reported for systemic *R. equi* disease and contextualize field observations.

For visual documentation, Figure 4 presents clinical photographs of the foal, showing bilateral corneal opacity and swelling of the carpal and hock joints. These manifestations are compatible with osteoarticular involvement described for severe *R. equi* disease in foals, including septic arthritis and osteomyelitis, which are associated with poor prognosis (19). Specifically, Ruocco et al. reported an 84% mortality rate in foals diagnosed with joint sepsis and osteomyelitis caused by *R. equi*, highlighting the critical importance of early diagnosis and intervention.

This illustrative case complements the sero-epidemiological findings and underscores the value of integrating clinical observation with laboratory testing in foal populations under similar management conditions. It highlights the importance of combining laboratory diagnostics with clinical vigilance and reinforces the designated necessity for early case detection and intervention strategies in foal populations under similar pastoral management systems.

### 3.4 Isolation and molecular confirmation of *Rhodococcus equi*

**Figure 5:**
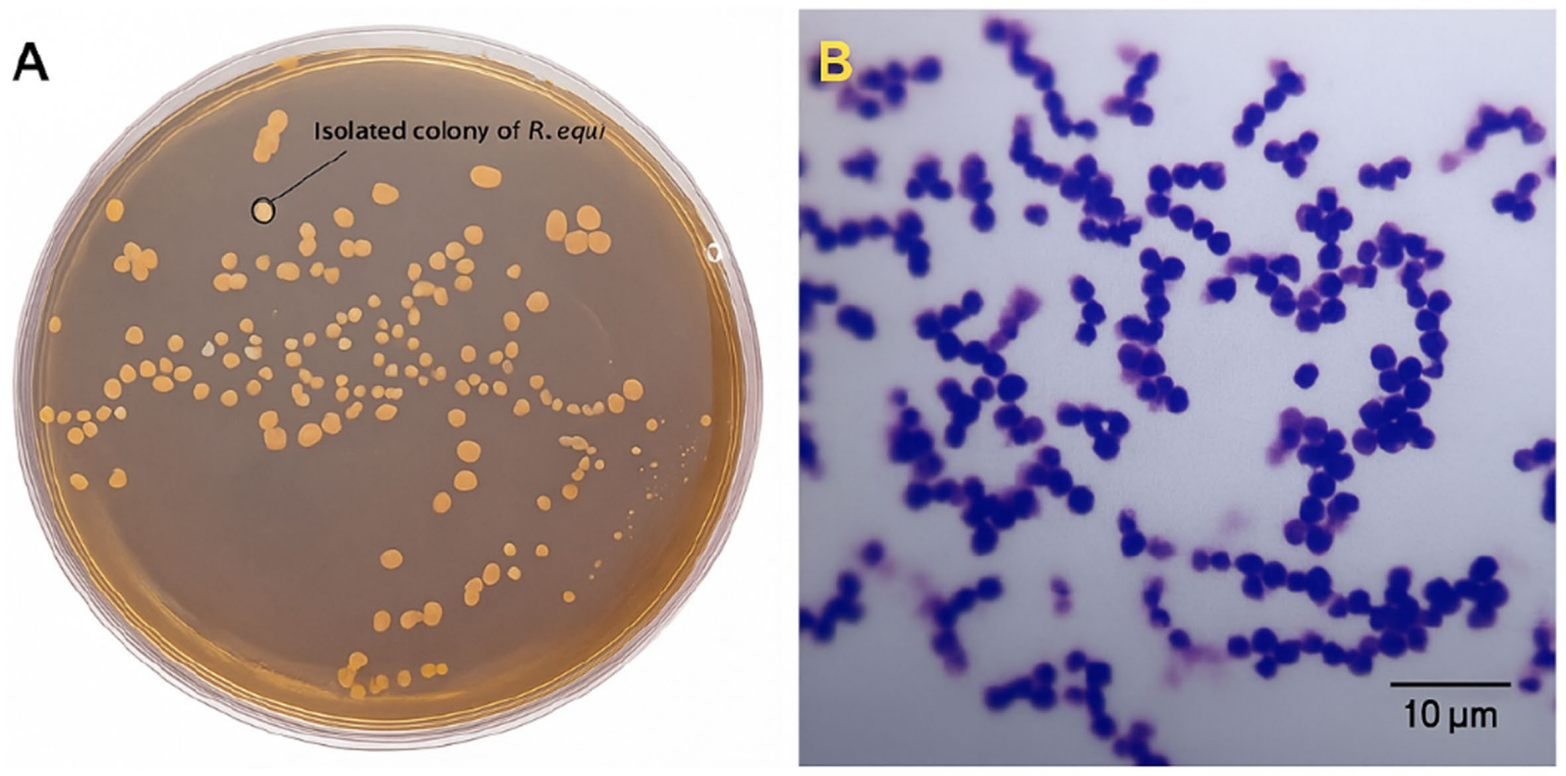
Colony and microscopic morphology of *Rhodococcus equi*. **(A)** Isolated colonies of *R. equi* grown on Columbia blood agar after 48 h at 37 °C. Photograph taken under laboratory lighting conditions. **(B)** Gram-stained *R. equi* cells visualized by light microscopy (Olympus CX43, 100 × oil immersion objective). Scale bar = 10 μm.

To complement serological findings and provide microbiological evidence consistent with pathogen circulation, microbiological culture and molecular identification were performed on clinical samples obtained from foals with systemic signs or found deceased at three farms. A total of seven specimens (lung tissue, tracheobronchial lymph nodes, nasal swabs) were collected aseptically. After 48–72 h of aerobic incubation at 37 °C, mucoid, salmon-pink colonies presumptively consistent with *R. equi* were recovered from three samples: two necropsied foals (foal A: lung; Foal B: tracheobronchial lymph node) and one clinically affected foal (nasal swab). Both carcasses showed gross changes compatible with rhodococcosis, including cranioventral pulmonary consolidation and enlarged tracheobronchial lymph nodes. Gram staining revealed Gram-positive coccobacilli in clusters, consistent with *R. equi* (Figure 5).

Presumptive identification based on colony morphology and Gram staining was supported by biochemical profiling: catalase-positive, urease-positive, with a synergistic CAMP reaction with *Staphylococcus aureus*, consistent with the established phenotype of *R. equi*. Results are summarized in Table 2.

**Table 2:** Biochemical and molecular characteristics of *R. equi* isolates.

Isolate ID	Source (Tissue)	Region (Farm)	Catalase	Urease	CAMP Reaction	16S rRNA PCR	BLASTn Identity
RE-KZ-1	Lung	Talgar (Farm 17)	+	+	Positive	~800 bp	99.8%
RE-KZ-2	Lymph node	Talgar (Farm 17)	+	+	Positive	~800 bp	99.6%
RE-KZ-3	Lung	Zhambyl (Farm 18)	+	+	Positive	~800 bp	99.9%

**Figure 6:**
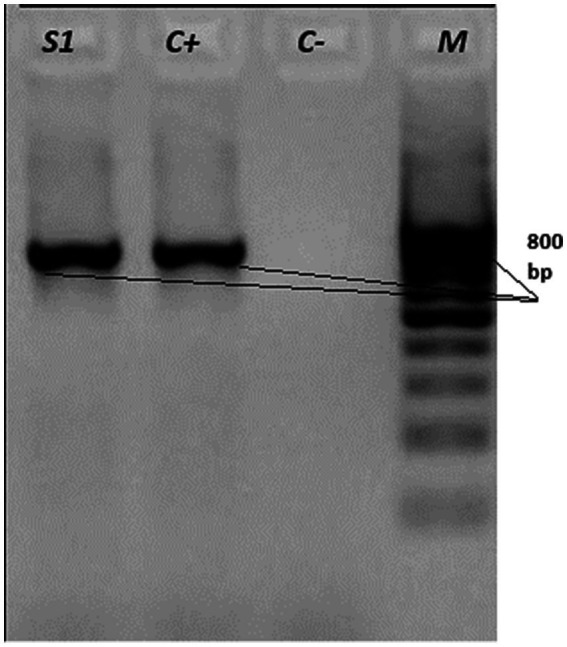
Agarose gel electrophoresis of PCR products targeting the 16S rRNA gene of *Rhodococcus equi*. Lanes: S1 - field sample; C + − positive control; C− − negative control; M - molecular weight marker (100–1,000 bp, 100 bp step). The presence of the expected band in the sample and positive control confirms successful amplification.

To genetically verify the isolates, genomic DNA was extracted from pure cultures and the 16S rRNA gene was amplified using universal bacterial primers as described in §2.6. PCR products were visualized by agarose gel electrophoresis (Figure 6) and sequenced by Sanger (ABI 3500). After quality trimming, a high-quality ~800 bp consensus region was retained for downstream analyses. BLASTn showed ≥99% identity to validated *R. equi* sequences in GenBank. A representative sequence has been deposited under GenBank accession OP448586.

This successful isolation and sequence-based confirmation of viable *R. equi* from clinical field cases provides, to our knowledge, among the first molecularly characterized equine isolates reported from Kazakhstan. Comparative genomic work on *R. equi* highlights substantial core and accessory gene content, including virulence and antimicrobial resistance elements; these findings motivate future whole-genome characterization of Kazakhstani isolates (20). The availability of this strain provides a valuable reference for future genomic, virulence factor, and antimicrobial resistance studies in the region.

### 3.5 Phylogenetic analysis

**Figure 7:**
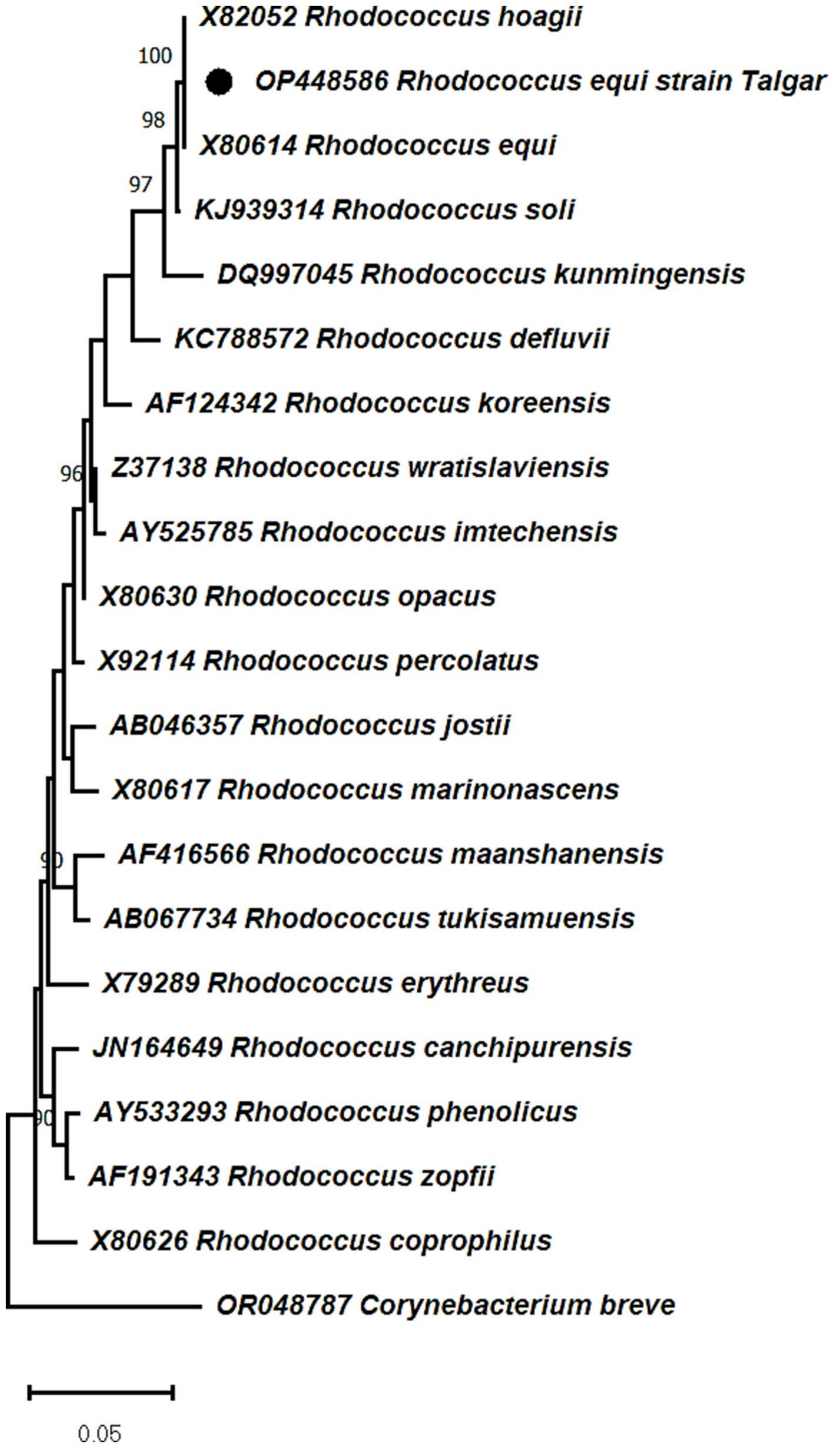
Neighbor-joining phylogeny (K2P distances; 1,000 bootstrap replicates) based on partial 16S rRNA sequences. *Corynebacterium breve* (OR048787) was used as an outgroup from the same family. The Kazakhstani isolate (OP448586) clusters within the *R. equi* clade, confirming species-level identity; within this clade it groups with equine-associated isolates from Germany, Japan, and the United States. Phylogenetic distance is shown by the scale bar, and bootstrap values (≥70) are indicated at the nodes.

To investigate the placement of the Kazakhstani *R.equi* isolate and its relatedness to globally circulating strains, we analyzed partial 16S rRNA sequences. The quality-trimmed consensus (~800 bp) from a confirmed field isolate (GenBank OP448586) was aligned with 25 reference *R. equi* sequences from GenBank representing diverse host origins (equine, porcine, bovine, human, environmental) and geographic regions (Europe, North America, East Asia, Middle East). Multiple sequence alignment used Clustal Omega; phylogeny was inferred by neighbor-joining (MEGA X) with Kimura two-parameter distances and 1,000 bootstrap replicates. The resulting tree is shown in Figure 7.

The tree was constructed using the neighbor-joining method (K2P distances) in MEGA X. The Kazakhstani isolate clusters within the *R. equi* clade, supporting its taxonomic identity and placement, in agreement with previous genomic and phylogenomic classifications of the genus *Rhodococcus*. The sequence groups with equine-associated *R. equi* reported from Germany, Japan, and the United States (sequence identity ≥99.5%). As 16S rRNA provides limited resolution for virulence assessment (21, 22), phylogenetic relatedness to equine lineages cannot establish pathogenic potential or zoonotic risk. Nevertheless, the grouping of the Kazakhstani sequence with equine-associated isolates aligns with the clinical suspicion of rhodococcosis and the serological findings reported in this study. To our knowledge, this represents one of the first phylogenetically characterized equine *R. equi* isolates from Kazakhstan deposited in a public database, underscoring the need for broader molecular surveillance across Central Asia. In contrast, *R. equi* isolates from pigs and environmental sources formed distinct branches, suggesting host-associated divergence within the species.

## 4 Discussion

To our knowledge, this is among the first integrated sero-epidemiological and molecular investigations of *R. equi* in horses in Kazakhstan and provides initial region-specific insights into exposure and molecular identity. While *R. equi* has recognized relevance for immunocompromised humans, our study did not assess zoonotic transmission. The combination of ELISA-based serosurveillance, microbiological isolation, and 16S rRNA-based phylogenetic analysis offers a comprehensive picture of *R. equi* circulation among horses in different regions of the country.

The overall seroprevalence of 8.3% detected in this study aligns with findings reported in several countries with endemic *R. equi* infection. Similar prevalence rates have been described in west northern Rajasthan, India – 9% (23), in Germany – 3% (24), in Turkey – 11,1% (25), with a report from Israel also showing comparable seroprevalence dynamics in foals and adults (26). *R. equi* exposure in Kazakhstan is consistent with patterns observed in regions with established equine industries. Notably, the significantly higher seropositivity among foals (25%) compared to adult horses (5.9%) is consistent with the known age-dependent susceptibility due to immature cell-mediated immunity and perinatal exposure to contaminated environments (27). Consistent with this biology, foals showed greater dispersion of OD values, with a subset of high-titer responders. This variability is plausibly driven by heterogeneity in timing of exposure within the 1–6-month.

Between-farm heterogeneity with higher foal seropositivity at several farms in the Almaty region suggests localized clusters of exposure. Given small cell counts at some farms, we emphasize point estimates with 95% CIs and use exact tests where appropriate; not all contrasts reached statistical significance. We did not assess plasmid virulence markers, therefore we refer to circulation of *R. equi* rather than “virulent lineages”.

Isolation of *R. equi* from three clinical samples complements the seroepidemiological findings and provides material for regional strain characterization. Colony morphology and Gram-stain features, together with the biochemical profile (catalase-positive, urease-positive, synergistic CAMP with *Staphylococcus aureus*), were consistent with *R. equi*. PCR amplification and Sanger sequencing of the 16S rRNA gene yielded a quality-trimmed consensus (~800 bp) showing ≥99% identity to validated *R. equi* sequences by BLASTn, supporting species-level identification; virulence plasmid markers (e.g., *vapA/vapB*) were not assessed. The sequence has been deposited in GenBank (OP448586) and represents, to our knowledge, among the first molecularly characterized equine *R. equi* isolates reported from Kazakhstan.

Phylogenetic analysis showed that the Kazakhstani isolate clusters, with strong bootstrap support, with equine-associated *R. equi* sequences from Germany, Japan, and the United States. This relatedness is consistent with the broad geographic distribution of equine-associated *R. equi*; however, 16S rRNA has limited resolution for inferring virulence or transmission routes, and plasmid markers (e.g., *vapA/vapB*) were not assessed here. The separation of porcine and environmental *R. equi* sequences is compatible with host-associated structure within the species, but the phylogeny does not by itself establish the source of infection in this case.

From a One Health perspective, the detection of *R. equi* in equine populations is relevant given its opportunistic potential in immunocompromised humans. The relevance of sero-epidemiological investigations for zoonotic and emerging pathogens in livestock has also been demonstrated in Kazakhstan through serological evidence of exposure to MERS or MERS-like coronaviruses in Bactrian and dromedary camels (28). Although zoonotic transmission appears uncommon, *R. equi* has been documented as a cause of severe pulmonary infection in HIV/AIDS patients and organ-transplant recipients. Virulence plasmids encoding Vap proteins have been identified in *R. equi* isolates from AIDS patients in Cuba, suggesting overlap with equine-associated lineages (29). Additional clinical cases in immunocompromised individuals, including renal-transplant recipients, have been reported by Kanguli et al. (30). The emergence of antibiotic-resistant *R. equi* in veterinary and clinical settings further underscores the need for risk-based surveillance and prudent antimicrobial use (31). Recent study on the pathogenesis and immune responses of indigenous cattle experimentally infected with lumpy skin disease virus have provided valuable insights into clinical outcomes and antibody kinetics (32). *In vitro* studies indicate that, among tested macrolides, clarithromycin showed comparatively strong activity against *R. equi*, particularly in combination with rifampicin or doxycycline at concentrations relevant for foal therapy (33). We note that our study did not evaluate human cases, antimicrobial susceptibility, or virulence plasmids; thus, these observations provide context rather than direct evidence from the present dataset.

In this context, the results of this study have practical implications for risk-based monitoring and farm-level biosecurity in Kazakhstan. The higher seropositivity observed in foals, together with isolation of *R. equi* from clinical cases, suggests that early-life environmental management (e.g., dust control, manure handling, housing hygiene) may be important for reducing exposure (34). Incorporation of *R. equi* screening into routine health monitoring of foals - particularly on large stud farms - could be considered, using validated assays where available, to support earlier recognition and management.

Finally, the findings lay the groundwork for future studies on antimicrobial resistance, virulence-plasmid gene carriage (e.g., *vapA*), and whole-genome sequencing of local *R. equi* to better characterize epidemiologically relevant features and inform targeted control strategies (35). Additional vaccine candidates targeting alternative virulence-associated proteins, such as *VapG*, have shown partial protection in murine models, highlighting the potential for novel immunogens (36). Recent seroepidemiological work in other species (e.g., goats) underscores the importance of distinguishing *R. equi* lineages by plasmid type, especially *vapN*-associated strains (37).

## 5 Conclusion

To our knowledge, this study provides among the first integrated serological and molecular data on *R. equi* in horses from three regions of Kazakhstan, indicating circulation and higher exposure among foals. We isolated *R. equi* from clinical cases, and 16S rRNA–based identification with phylogenetic analysis supported species-level identity and grouping with equine-associated isolates from Europe, Japan, and North America. These findings are consistent with a One Health perspective, although zoonotic transmission was not assessed in this study.

Given that 16S rRNA has limited resolution for inferring virulence and plasmid markers (e.g., *vapA/vapB*) were not typed, our results should be interpreted as a baseline for the region. Priorities include risk-based monitoring, farm-level biosecurity, and targeted research on virulence determinants and antimicrobial susceptibility of local isolates, alongside validation of serological assays and broader geographic/environmental sampling. This work lays the groundwork for evidence-based prevention and underscores the value of integrating veterinary microbiology and molecular epidemiology into national and regional health policies.

## Data Availability

The datasets generated for this study, including the *Rhodococcus equi* 16S rRNA gene sequence, are available in the GenBank repository under accession number OP448586. Additional raw data supporting the conclusions of this article will be made available by the authors upon reasonable request.

## Supplementary material

SUPPLEMENTARY FIGURE S1 Estimated global distribution of horse populations by country in 2025. The map illustrates relative population sizes, with darker colors indicating higher horse densities.

Supplementary FIGURE S2 Histogram of ELISA optical density (OD) values from serum samples. The distribution is accompanied by descriptive statistics, including minimum, maximum, mean, median, and quartiles.

Supplementary FIGURE S3 Distribution of samples across predefined ELISA OD ranges. Box plots show medians, interquartile ranges, and outliers, providing an overview of variation among categories.

Supplementary Table S1 Average ELISA optical density (OD) values by farm and location, presented with 95% confidence intervals. The table summarizes group-level variation and supports comparison across sampling sites.
