# Correction: Integrated molecular and serological survey of *Rhodococcus equi* in horses from three regions of Kazakhstan

**DOI:** 10.3389/fvets.2025.1734084

**Published:** 2025-11-17

**Authors:** Makpal Zanilabdin, Gulnaz Ilgekbayeva, Bauyrzhan Otarbayev, Raikhan Nissanova, Gulzhan Mussayeva, Shinji Takai, Yasunori Suzuki, Tsutomu Kakuda, Serikzhan Kurman, Yerken Kassymov, Bayan Valiyeva

**Affiliations:** 1 Kazakh National Agrarian Research University, Faculty of Veterinary and Zooengineering, Almaty, Kazakhstan; 2 Virology Laboratory, Kazakh Scientific Research Veterinary Institute, Almaty, Kazakhstan; 3 National Veterinary Reference Center, Almaty, Kazakhstan; 4 School of Veterinary Medicine, Kitasato University, Towada, Japan; 5 Tokyo University of Agriculture and Technology, Fuchu, Japan

**Keywords:** *Rhodococcus equi*, seroprevalence, Kazakhstan, zoonotic pathogen, phylogenetic analysis, equine respiratory disease, molecular epidemiology

An incorrect grant number was provided for the Ministry of Science and Higher Education of the Republic of Kazakhstan in the **Funding** statement.

The correct number is AP19680565.

The original version of this article has been updated.

