# Supplementary figures and images for: Integrated molecular and serological survey of Rhodococcus equi in horses from three regions of Kazakhstan
Source: Front Vet Sci. 2025 Oct 21;12:1650186. doi: 10.3389/fvets.2025.1650186 (PMC12584071; doi:10.3389/fvets.2025.1650186)

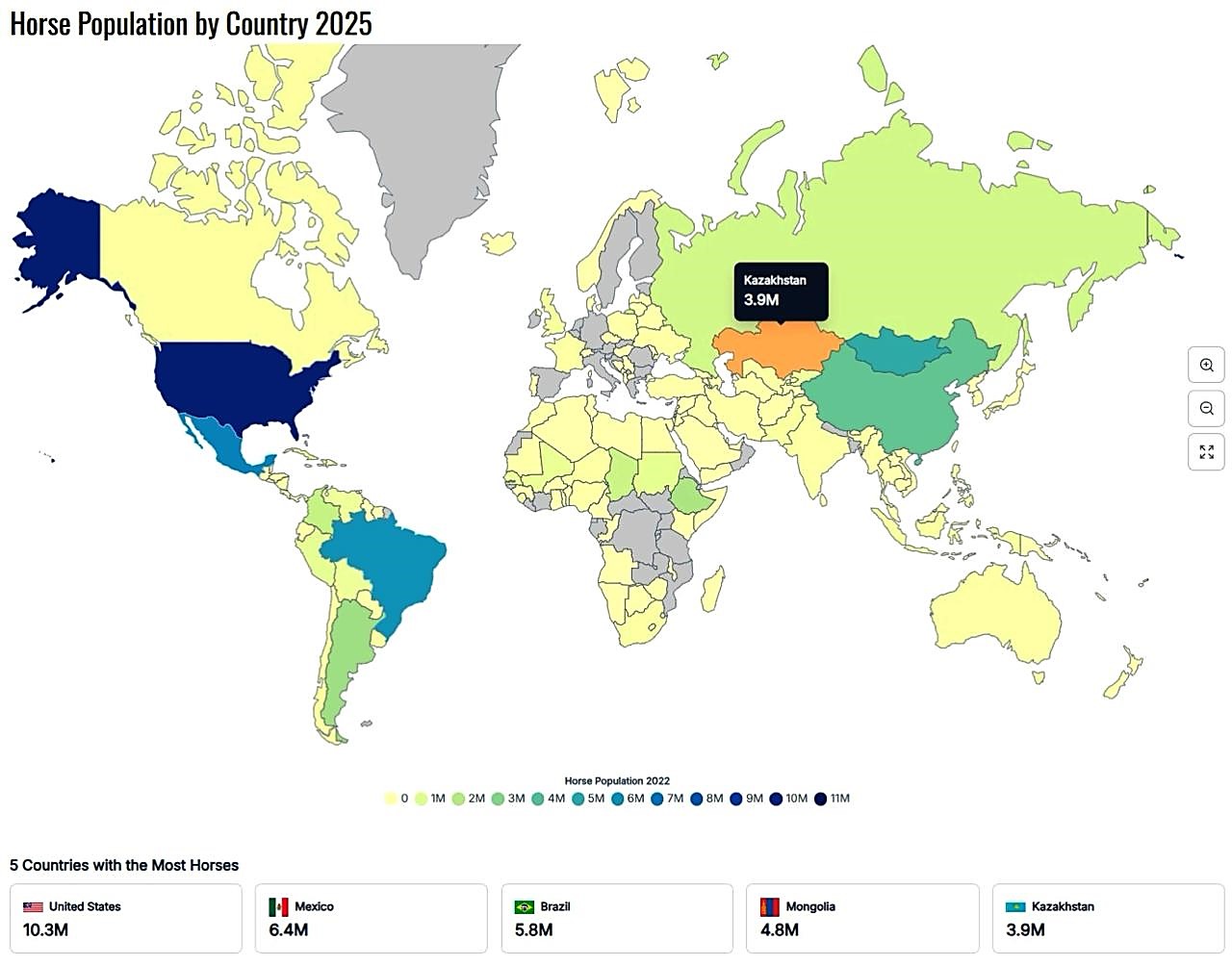

Supplement: SUPPLEMENTARY FIGURE S1 — Estimated global distribution of horse populations by country in 2025. The map illustrates relative population sizes, with darker colors indicating higher horse densities. [file Image_1.jpeg]

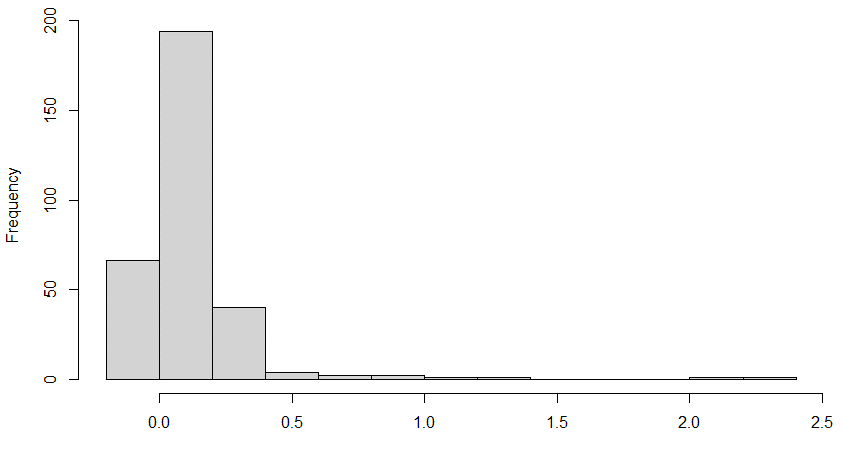

Supplement: Supplementary FIGURE S2 — Histogram of ELISA optical density (OD) values from serum samples. The distribution is accompanied by descriptive statistics, including minimum, maximum, mean, median, and quartiles. [file Image_2.png]

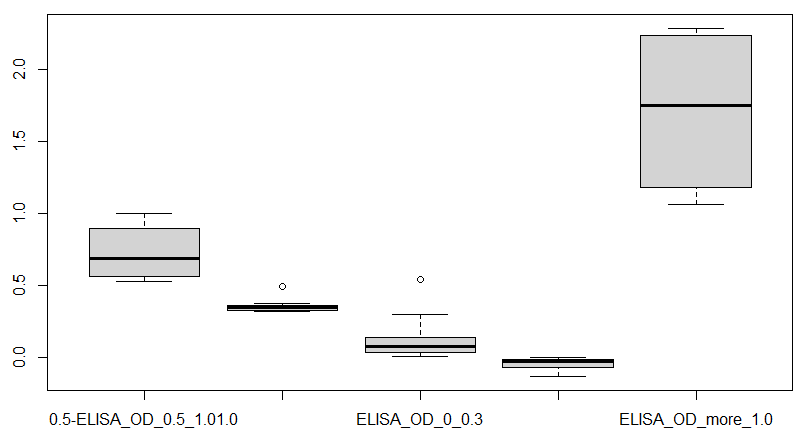

Supplement: Supplementary FIGURE S3 — Distribution of samples across predefined ELISA OD ranges. Box plots show medians, interquartile ranges, and outliers, providing an overview of variation among categories. [file Image_3.png]

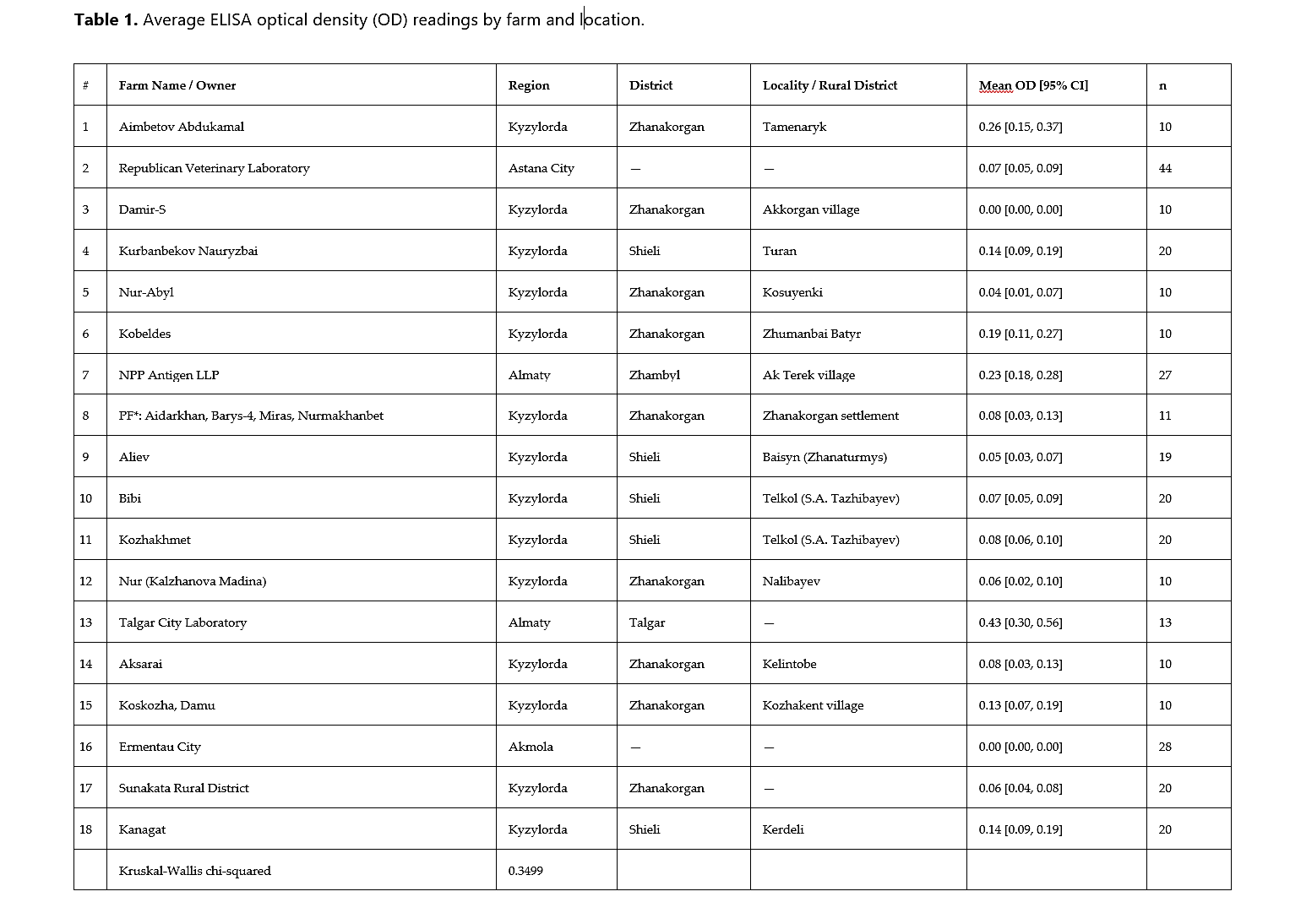

Supplement: Supplementary Table S1 — Average ELISA optical density (OD) values by farm and location, presented with 95% confidence intervals. The table summarizes group-level variation and supports comparison across sampling sites. [file Supplementary_file_1.png]
